# Discovering and validating unknown phospho-sites from p38 and HuR protein kinases *in vitro *by Phosphoproteomic and Bioinformatic tools

**DOI:** 10.1186/2043-9113-1-16

**Published:** 2011-07-06

**Authors:** Elena López, Isabel López, Julia Sequí, Antonio Ferreira

**Affiliations:** 1 Phosphoproteomic core, Spanish National Cancer Research Centre (CNIO), C/Melchor Fernández Almagro, 3, 28029, Madrid, Spain; 2 Hematology Department, Hospital QUIRÓN, Madrid, Diego de Velázquez 1, 28223, Pozuelo, Madrid, Spain; 3 Immunology Department, Hospital Carlos III, Sinesio Delgado 28029, Madrid, Spain; 4 Immunology Department, Hospital Universitario La Paz, P° de la Castellana 261-28046, Madrid, Spain; 5 Inflammatory core, Centro de Investigación i+12 del Hospital Universitario 12 de Octubre, Avda de Córdoba s/n 28041, Madrid, Spain

## Abstract

**Background:**

The mitogen activated protein kinase (MAPK) pathways are known to be deregulated in many human malignancies. Phosphopeptide identification of protein-kinases and site determination are major challenges in biomedical mass spectrometry (MS). P38 and HuR protein kinases have been reported extensively in the general principles of signalling pathways modulated by phosphorylation, mainly by molecular biology and western blotting techniques. Thus, although it has been demonstrated they are phosphorylated in different stress/stimuli conditions, the phosphopeptides and specific amino acids in which the phosphate groups are located in those protein kinases have not been shown completely.

**Methods:**

We have combined different resins: (a) IMAC (Immobilized Metal Affinity Capture), (b) TiO_2 _(Titanium dioxide) and (c) SIMAC (Sequential Elution from IMAC) to isolate phosphopeptides from p38 and HuR protein kinases *in vitro*.

Different phosphopeptide MS strategies were carried out by the LTQ ion Trap mass spectrometer (Thermo): (a) Multistage activation (MSA) and (b) Neutral loss MS3 (DDNLMS3).

In addition, Molecular Dynamics (MD) bioinformatic simulation has been applied in order to simulate, over a period of time, the effects of the presence of the extra phosphate group (and the associated negative charge) in the overall structure and behaviour of the protein HuR.

This study is supported by the Declaration of Helsinki and subsequent ethical guidelines.

**Results:**

The combination of these techniques allowed for:

(1) The identification of 6 unknown phosphopeptides of these protein kinases. (2) Amino acid site assignments of the phosphate groups from each identified phosphopeptide, including manual validation by inspection of all the spectra. (3) The analyses of the phosphopeptides discovered were carried out in four triplicate experiments to avoid false positives getting high reproducibility in all the isolated phosphopeptides recovered from both protein kinases. (4) Computer simulation using MD techniques allowed us to get functional models of both structure and interactions of the previously mentioned phosphorylated kinases and the differences between their phosphorylated and un-phosphorylated forms.

**Conclusion:**

Many research studies are necessary to unfold the whole signalling network (human proteome), which is so important to advance in clinical research, especially in the cases of malignant diseases.

## Introduction

As with other MAPK pathways, the p38 signalling cascade involves sequential activation of MAPK kinase kinases (MAP3Ks) and MAPK kinases (MKKs) including MKK3, MKK4, and MKK6, which directly activate p38 through phosphorylation in a cell-type- and stimulus-dependent manner [1,2]. Once activated, p38 MAPKs phosphorylate serine/threonine residues on their substrates, such as transcription factors, cell cycle regulators as well as protein kinases. The p38 signalling pathway allows cells to interpret a wide range of external signals, such as inflammation, hyperosmorality, oxidative stress and respond appropriately by generating a plethora of different biological effects [3-14]. HuR has been implicated in processes such carcinogenesis, proliferation, immune function or responsiveness to DNA damage [15].

It is of interest to note that numerous HuR-regulated mRNAs encode proteins responsible for implementing five major cancer traits:

(a) Promote cell proliferation (p27, cyclin D, Cyclin E1 or EGF)

(b) Increase cell survival (SIRT1, Mdm2 or p21)

(c) Elevate local angiogenesis (VEGF, Cox-2 or HIF-1alpha)

(d) Invasion and metastasis (Snail, MMP-9, or uPA)

(e) Evasion of immune recognition (TGF-beta).

Moreover, HuR was broadly elevated in cancer tissue compared to the corresponding non-cancer tissues. It has been widely reported that in the general principles of signalling pathways p38 and HuR kinases are modulated by phosphorylation, mainly by western blotting techniques. The phosphopeptides and the specific amino acids in which the phosphate groups are located in these low expressed proteins have not been completely shown as yet [16-22].

The analysis of the spatial and temporal aspects of protein phosphorylation is of great interest for the discovery of functions of specific biological processes. An extensive mass spectrometry-based mapping of the phosphoproteome progresses and computational analysis of phosphorylation has been carried out. Phosphorylation-dependent signalling becomes increasingly important for clinical research and requires improvements for each different sample. In addition, the linear sequence motifs that surround phosphorylated residues have been successfully used to characterize kinase-substrate specificity. To complement phosphoproteomic research, bioinformatics offers a range of methods to analyze and to simulate structural properties of the studied phosphoproteins. Both unphosphorylated and phosphorylated states of a residue can be generated "in silico" and included in the appropriate 3D protein context. After this initial modelling, Molecular Dynamics (MD) techniques can be applied in order to simulate, over a period of time, the effects of the presence of the extra phosphate group (and the associated negative charge) in the overall structure and behaviour of the protein [23-25].

We describe the successful strategy (also used by other scientists [26-28]) for the discovery of 6 unknown phosphorylated peptides from p38 and HuR kinases. Our data comes from advances in MS strategies coupled to different resins (IMAC, TiO_2 _and SIMAC) that we have applied, coupled to bioinformatics tools (MD simulation). The specific peptides discovered, which are phosphorylated in p38 and HuR protein kinases, are provided. In addition, the specific amino acid assignments of the phosphate groups from the identified phosphopeptides are also presented. Unknown phospho-sites from these kinases *in vitro *have been discovered for the first time. Our data is supported by previous scientific studies related to these protein phosphorylated kinases.

It has have been reported that p38 and HuR kinases are phosphorylated mainly by western blotting techniques although not showing all amino acids in which the phosphate groups are located. It should be pointed out that the phosphate groups can vary according to the conditions of the sample analysis (see references of p38 and HuR previously mentioned [16-22]). In this study, MSA (multistage activation) compared to DDNLMS3 (neutral loss MS3) gave more information for the suite of phosphopeptides studied when using SIMAC coupled to the ion Trap mass spectrometer. Using bioinformatics MD simulations we have proposed functional variations in both structure and interactions of the previously mentioned phosphorylated-kinases comparing the phosphorylated and un-phosphorylated forms previously described *in vitro*. Finally, we point out possible developments or alternatives and complementary tools with the intention of providing the community with improved and additional phosphorylation studies of cellular signalling networks, this being such an important issue owing to the fact that if we had complete knowledge of the signalling-networks, many malignant diseases could be more fully understood and thus facilitate drug development for different pathologies. This article also aims to improve the knowledge of p38 and HuR protein kinases by identifying and validating new phosphopetides *in vitro*, with the knowledge that this is essential to advance in the knowledge of signalling networks (human proteome). These and many other advances will help clinical research investigations, especially in relation to human malignant diseases.

## Materials and methods

### Statement of ethical approval

This study was conducted in compliance with the international "Declaration of Helsinki." An informed consent about the procedures as well as permission from the Ethical Committee of Carlos III Hospital of Health was obtained. This study adhered to the tenets of the Declaration of Helsinki. (http://www.wma.net/e/policy/b3.htm). (Declaration of Helsinki (1964), Belmont (1978) and agreement of Oviedo (1997) - the basic principles for human and biological samples research studies -) http://www.isciii.es/htdocs/index.jsp).(http://www.madrid.org/cs/Satellite?pagename=HospialCarlosIII, http://www.cnio.es "working links")

### Purification and Kinase assay

Recombinant glutathione S-transferase (GST) fusion proteins were expressed in Escherichia coli BL21 (DE3) and purified using standard protocols. p38beta was activated with MalE-MKK6DD (5:1 ratio) in 50 mM Tris-HCl, pH 7.5, 10 mM MgCl2, 2 mM DTT pH 7.5 and 200 uM ATP for 1 hour at 30°C. Kinase assay were carried out in a buffer A (50 mM Tris-HCl, pH 7.5, 10 mM MgCl_2_, 2 μM microcystin, 50 mM NaF, and 10 μM ATP) supplemented with Phosphatase inhibitor cocktail 1 (P2850, 1:100) and Phosphatase inhibitor cocktail 2 (P5726, 1:100) from SIGMA, containing 12 μg of HuR and 500 ng of activated p38 for 30 min at 30°C.

### Protein digestion in solution

Proteins (10 μg) were subjected to digestion procedure following the protocol described by Zhao and co-workers with slight variations [29]. Digestion with Lysyl Endopeptidase: the reduced and alkylated sample was incubated at room temperature for 3 h with 1 μg of lysyl endopeptidase/50 μg protein (WAKO). Digestion with Trypsin: the lysyl endopeptidase-digested sample was diluted with 50 mM NH_4_HCO_3 _(Sigma) to make a 5 times dilution of urea, since trypsin is not fully active at high concentrations of urea. One microgram of modified trypsin (Promega) was added per 50 μg of lysyl endopeptidase-digested protein and the sample was incubated at room temperature for 16-24 h. The digests were evaporated to about 20 μL in a SpeedVac centrifuge and subsequently 5 μl were used for TiO_2_, 5 μl for IMAC and 5 μl for SIMAC phosphopeptide enrichments.

### Dioxide Titanium phosphoenrichment (TiO_2_)

Titanium dioxide-microcolumns with a length of ~2 mm were packed in GELoader tips. A small plug of C8 material was stamped out of a 3M Empore C8 extraction disk using an HPLC syringe needle and placed at the constricted end of the GELoader tip. The C8 disk serves only as a frit to retain the titanium dioxide beads within the GELoader tip.

Note that the solvent used for either washing or loading the sample onto the TiO_2 _microcolumn contains organic solvent (50-80% CH_3_CN), which abrogates adsorption of peptides to the C8 material. The TiO_2 _beads were suspended in 80% acetonitrile, 0.1% TFA, and an aliquot of this suspension (depending on the size of the column) was loaded onto the GELoader tip. Gentle air pressure created by a plastic syringe was used to pack the column as described previously. The bound peptides were eluted using 3 μl of NH4OH, pH 10.5. An additional elution step using 0.5 μL of 30% acetonitrile was added to elute peptides, which had remained bound to the C8 membrane plug. The eluents were pooled and acidified using 100% formic acid prior to the desalting step and desalted using Poros-R3 coupled to C18-Disks microcolumns prior to MS analysis [30,31].

### Immobilized Metal Affinity Capture (IMAC) phosphoenrichment

Purification of phosphorylated peptides was performed according to Nuhse and co-workers [32] and Lee and co-workers with minor changes [33]. Briefly 10 μl of ironcoated PHOS-selectTM metal chelate beads (Sigma) were washed twice in 100 μl of washing/loading solution (0.25 M acetic acid, 30% acetonitrile) and resuspended in 40 μl of washing/loading solution. An aliquot of this solution (20 μl) was incubated with the peptide solution in a total volume of 40 μl of washing/loading solution for 30 min with constant rotating. After incubation, the solution was loaded onto a constricted GELoader tip, and gentle air pressure was used to pack the beads. Subsequently the beads were washed extensively with the washing/loading solution. The bound peptides were eluted using 3 μl of NH_4_OH, pH 10.5, and desalted using Poros R3 coupled to C18-Disks microcolumn prior to MS analysis.

### Sequential Elution from IMAC (SIMAC) phosphoenrichment

For each experiment 10 μl of iron-coated PHOS-selectTM metal chelate beads IMAC (Sigma) were used. The beads were washed twice in loading buffer (0.1% TFA, 50% acetonitrile) as described previously [34]. The beads were incubated with 30 μl of loading buffer and 4 μg of peptide mixture (tryptic digest). The beads were shaken in a Thermomixer (Eppendorf) for 30 min at 20°C. After incubation, the beads were packed in the constricted end of a 200 μl GELoader tip (Alpha Laboratories) by application of air pressure forming an IMAC microcolumn. The IMAC flow-through was collected in an Eppendorf tube for further analysis by TiO_2 _chromatography (see below). The IMAC column was washed using 20 μl of loading buffer, which was pooled with the IMAC flow-through. The putative monophosphorylated peptides and contaminating non-phosphorylated peptides were eluted from the IMAC column using 10 μl of 1% TFA, 20% acetonitrile, and the possible multiple phosphorylated peptides were subsequently eluted from the same IMAC microcolumn using 40 μl of ammonia water, pH 11.3 (10 μl of 25% ammonia solution (Merck) in 490 μl of ultra-high quality water). The IMAC flow-through and the IMAC eluents were dried by lyophilization. Titanium Dioxide (TiO_2_) Chromatography after lyophilization, the pooled flow-through and wash from the IMAC microcolumn was enriched for phosphopeptides using TiO_2 _chromatography. For the complex mixture of the putative monophosphorylated peptide fraction (1% TFA) was also subjected to TiO_2 _chromatography as described below. A TiO_2 _microcolumn was prepared by stamping out a small plug of C8 material from a 3M EmporeTM C8 extraction disk (3M Bioanalytical Technologies) and placing the plug in the constricted end of a P10 tip (Eppendorff). The TiO_2 _beads (suspended in 100% acetonitrile) were packed in the P10 tip where the C8 material prevented the beads from leaking. The TiO_2 _microcolumn was packed by the application of air pressure. Buffers used for loading or washing of the microcolumn contained 80% acetonitrile to prevent non-specific binding to the C8 membrane and the TiO_2 _beads. The lyophilized sample was resuspended in 2 μl of 4 M urea and 3 μl of 1% SDS and diluted five times in loading buffer (1 M glycolic acid (Fluka) in 80% acetonitrile, 5% TFA) and loaded onto a TiO_2 _microcolumn of 5 mm [35]. The TiO_2 _microcolumn was washed with 5 μl of loading buffer and subsequently with 30 μl of wash buffer (80% acetonitrile, 5% TFA). The phosphopeptides bound to the TiO_2 _microcolumns were eluted using 50 μl of ammonium water (pH 11.3) followed by elution using 0.5 μl of 30% acetonitrile to elute phosphopeptide bound to the C8 disk. The eluent was acidified by adding 5 μl of 100% formic acid prior to the desalting step.

### Desalting the isolated phosphopetides by chromatography reversed phase (RP) using POROs R3 coupled to C18 Disks, prior to MALDI and ESI Mass Spectrometry analysis

Poros Oligo R3 reversed phase material was from PerSeptive Biosystems (Framingham, MA). GELoader tips were from Eppendorf (Eppendorf, Hamburg, Germany) and Alpha Laboratories (Hampshire, UK). Orthophosphoric acid (85%, v/v) was from J. T. Baker Inc. Ammonia solution (25%) was from Merck. 3M Empore C8 disk was from 3M Bioanalytical Technologies (St. Paul, MN). All reagents used in the experiments were sequence grade, and the water was from a Milli-Q system (Millipore, Bedford, MA). The Poros Oligo R3 reversed phase resin (PerSeptive Biosystems) was dissolved in 70% acetonitrile. The R3 beads were loaded onto constricted GELoader tips, and gentle air pressure was used to pack the beads to obtain R3 microcolumns of 2 mm. Each acidified sample was loaded onto a R3 microcolumn. The R3 microcolumns were subsequently washed with 30 μl of 0.1% TFA, and the phosphopeptides were eluted directly onto the MALDI target using 0.5 μl of 20 μg/μl DHB (Fluka), 50% acetonitrile (ACN), 1% phosphoric acid. MALDI-MS analysis was just carried out in order to check there were sufficient eluted peptides to be analyzed by LC-ES-MS, after the microcolumns applied for the isolation, cleaning and concentration of *putative *phosphorylated peptides. For LC-ESI/MSMS analysis of the phosphorylated peptides originating from the sample, the phosphopeptides were desalted in a similar way; however, the phosphorylated peptides were eluted from the Poros R3 column coupled to C18 using 30 μl of 70% acetonitrile, 0.1% TFA followed by lyophilization. The phosphopeptides were subsequently resuspended in 0.5 μl of 100% formic acid and 10 μl of Buffer A (0.1% formic acid, and 5% ACN) prior to LC-ESI/MS^n ^analysis (see references previously mentioned [30,31,35]).

### Nano-LC-ESI-MSMS analysis using the LTQ ion Trap mass spectrometer

The nano-LC-MS experiments were performed using a LTQ ion Trap mass spectrometer (Thermo Electron, Bremen, Germany). The sample (5 μl) was applied onto an EASY nano-LC system following protocols from Thermo Company and Protein Research Group of Odense University courtesy. Each elute was then entered into a C18 reverse phase column (100 μm i.d., 10 cm long, 5 μm resin from Michrom Bioresources, Auburn, CA). The peptide mixtures were eluted with a 0-40% gradient (Buffer A, 0.1% formic acid, and 5% ACN; Buffer B, 0.1% formic acid and 95% ACN) over 180 min and were then online detected in LTQ ion Trap- mass spectrometer using a data-dependent TOP6 method. The general mass spectrometric conditions were: spray voltage, 1.85 kV; no sheath and auxiliary gas flow; ion transfer tube temperature, 1900C; 35% normalized collision energy using for MS/MS (MS2). Ion selection thresholds were: 500 counts for MS2. An activation q = 0.25 and activation time of 30 ms were applied in MS2 acquisitions. The mass spectrometer was operated in positive ion mode and a data-dependent automatic switch was employed between MS and MS/MS acquisition modes. For each cycle, one full MS scan in the LTQ ion Trap followed by ten MS2 in the LTQ at 5000 on the six most intense ions. Selected ions were excluded from further selection for 90 s. Maximum ion accumulation times were 1000 ms for full MS scans and 120 ms for MS2 scans. For the pseudo- MS3 method or Multi Stage Activation (MSA), an MSA was triggered if in the MS2 a neutral loss peak at -49, -32.7 or -24.5 Da was observed and that peak was one of the five most intense ions of the MS2 spectrum. To improve the fragmentation of phosphopeptides, multi-stage activation (MSA) in the Xcalibur software was enabled for each MS/MS spectrum. When a neutral loss of 97.97, 48.99, or 32.66 Thomson (Th) was detected, the MSA was applied to further fragment the ions. For the Neutral Loss MS3, MS Conditions were: the NanoMate^® ^100 was mounted to the Finnigan LTQ, and 5 μL (like for MSA) samples were infused at a rate of approximately 100 nL/min. Mass Spectrometer: Finnigan LTQ ion Trap. Ionization Mode: Nano-electrospray, Ion Polarity: Positive, Spray Voltage: 1.55 kV, Spray Pressure: 0.2 psi., Capillary Temperature: 150°C, Normalized Collision Energies: 20-25% for MSn., Maximum Scan Time: 50 ms., Number of Micro Scans Summed for Each Scan: 2-3. Neutral loss MS3 experiment activated for the loss of 98, 49 and 32.7 (singly, doubly and triply charged phosphopeptides). [Mascot searches (http://proteomicsresource.washington.edu/mascot/search_form_select.html) were carried out by "in-Mascot-house server of Centro Nacional de Investigaciones Oncológicas CNIO, http://www.cnio.es"].

### Database searching using an in-house MASCOT server and the validation of the identified phosphopeptides

The Mascot generic format file was produced by the following process: the utilities provided by Thermo Electron and Bioworks first converted Xcalibur binary (RAW) files into peak list (DTA) files, then the programs of merge.pl and merge.bat provided by MASCOT public web merged all DTA files into a Mascot generic format file.

For peptide or protein identification, all the raw data files were processed using BioWorks 3.3.1 (Thermo Finnigan, San Jose, CA) and the derived peak list was searched using the Mascot search engine (Matrix Science, London, UK) against a real and false human IPI protein database (V3.49), respectively. The following search criteria were employed: full tryptic specificity was required; two missed cleavages were allowed; carbamidomethylation (Cys) was set as fixed modification, whereas oxidation (Met), N-acetilation (protein), phosphorylation (STY), and intact phosphorylation (STY) were considered as variable modifications. Initial mass deviation of precursor ion and fragment ions was allowed up to 10 ppm and 0.5 Da, respectively. A peptide identified by Mascot was accepted if it had a peptide score above 20 in all the experiments performed. In addition, each phosphopeptide spectrum assignment was manually validated. All of the potential phosphopeptides were confirmed by manual interpretation of MS/MS and MSA ion spectra using the criteria described by Mann and Jensen [36], Gruhler and co-workers [37], and Thingholm and co-workers [38].

### Bioinformatics modelling and molecular dynamics simulations

**Figure 1:**
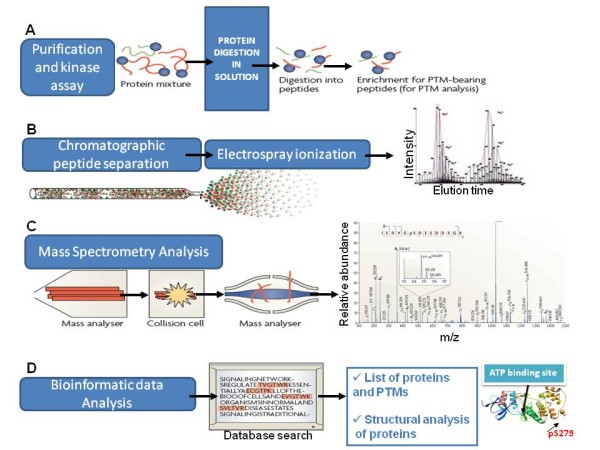
**The work flow for proteomic and bioinformatics PTM analysis is illustrated**. **[A] **Proteins isolated from kinase assays are in-solution digested into peptides using the proteases Lysyl Endopeptidase and Trypsin. The peptides containing specific post-translational modifications (phosphorylation) are enriched using different resins. Non-modified peptides are used to identify proteins. **[B] **Purified peptides are separated on a miniaturized reverse phase chromatography column with an organic solvent gradient. Peptides eluting from the column are ionized by electrospray at the tip of the column, directly in front of the mass spectrometer. **[C] **The electrosprayed ions are transferred into the vacuum of the mass spectrometer. In the mass spectrometer (MS mode) all ions are moved to the mass analyzer (ion Trap), where they are measured at high resolution. The mass analyser then selects a particular peptide ion and fragments it in a collision cell. For modified peptides, the peptide mass will be shifted by the mass of the modification, as will all fragments containing the modification, allowing the unambiguous placement of the PTM on the sequence. **[D] **The mass and lists of fragment masses for each peptide are scanned against protein sequence databases, resulting in a list of identified peptides and proteins. The lists of proteins and their peptides are the basis for bioinformatics analysis, in order to acknowledge improvements.

Crystal structure of MAP kinase p38beta protein and 3D coordinates of the quaternary structure of the first RNA recognition motif of human HuR protein were obtained from the Protein Data Bank (PDB <http://www.pdb.org> codes: 3GC8 - and 3HI9 -[39]- [40]-, respectively). As the published structure of HuR dimmer (dimmer) [40] showed a gap between residues 53 and 60 of the first monomer (chain B in 3HI9 structure), the coordinates for this external loop were completed by standard homology modelling procedures using the second monomer (chain D of 3HI9) as template. Model was built using SWISS-MODEL server facilities at http://swissmodel.expasy.org//SWISS-MODEL.html, and its structural quality was checked using the analysis programs provided by the same server (Anolea/Gromos/QMEAN4) [41-43]. Molecular dynamics (MD) simulations of the behaviour of human HuR dimmer (dimmer) in both unphosphorylated and phosphorylated states of Ser-48 residue were performed using the PMEMD module of AMBER10 and the parm-99 parameter set [44]. Two independent MD simulations were carried out: one for the modelled non-phosphorylated protein and a second one for the same system but containing a phosphorylated Ser residue in position 48. To simulate phospho-Ser, a tailored-made "prep" file for AMBER was used, as described in Mendieta and co-workers (2005) [45]. In order to neutralize the system's electrostatic charge, Cl^- ^counterions were placed in a shell around the system using a grid of coulombic potentials. The electrostatically neutralized complexes were then embedded in a truncated octahedron solvation box, keeping a distance of 12 Å between the limits of the box and the closest atom of the solute. Both counterions and solvent were added using the LEAP module of AMBER. Initial relaxation of each complex was completed by performing 10000 steps of energy minimization with a cut-off of 10.0 Å. Before starting the MD simulation, the temperature was raised from 0 to 298K, in a 200 ps continuous heating phase. During this stage, velocities were reassigned at each new temperature according to the Maxwell-Boltzmann distribution, and positions of the Cα trace of the solute were constrained with a force constant of 500 kcal mol^-1^rad^-2 ^to impede a spurious disorganization of the structure during the heating of the system. During the last 100 ps of the equilibration phase of the MD, the force constant was reduced stepwise down to 0 for all constrained atoms. Final trajectory length of both MD simulation processes were of 10 ns over the complete systems. During the full trajectory, SHAKE algorithm was used to constrain hydrogen bonds to their equilibrium values with an integration time step of 2 fs, updating the list of non-bonded pairs every 25 steps and saving coordinated every 2 ps. Periodic boundary conditions were applied. Electrostatic interactions were represented using the smooth particle mesh Ewald method with a grid spacing of about 1 Å. Final analysis of the trajectories was performed using the CARNAL module of AMBER10. The proteomics coupled to bioinformatics pipe-line strategy used for this research study of p38 and Hur protein kinases is illustrated (Figure 1).

## Results

### Identified phosphopeptides

(a) The aim of this study was to establish, as a routine path, a method for identification and characterization of individual phosphorylated kinases p38 and HuR *in vitro *using: TiO_2_, IMAC, SIMAC coupled to MSA and MS3NL on the LTQ ion Trap mass spectrometer (Thermo). Purification and fusion proteins were expressed in Escherichia coli. The kinase assay was carried out incubating with different types of protein phosphatase inhibitors in order to increase the levels of protein-kinases phosphorylation prior to the analysis. In fact, sodium pervanadate, a tyrosine phosphatase inhibitor, coupled to a combination of two phosphatase inhibitor cocktails from Sigma (one cocktail containing serine/threonine phosphatase inhibitors and one containing tyrosine phosphatase inhibitors) was also used. Protein kinases were digested with lysyl endopeptidase and trypsin and subsequently enriched for phosphorylated peptides using TiO_2_, IMAC and SIMAC phosphoenrichments. The isolated phosphopeptides were desalted, cleaned and analyzed by nano-LC ESI-MS/MS using a Thermo LTQ ion Trap MSMS instrument. All experiments were performed in triplicate. The LC-MS/MS experiments and Mascot database searching resulted in overall significant peptide hits. All the peptides were determined with a mass error of less than 5.5 ppm. A total of 6 phosphopeptides were validated by manual evaluation of the LC-MS/MS data sets obtained from the four triplicated experiments. Of these, 6 were assigned to unique amino acid phosphorylated sequences resulting in the identification of 3 unique proteins across all experiments.

(b) The analysis of the 5 μl (~3 μg) of the sample purified by IMAC and desalted and cleaned by R3/C18 permitted us to obtain 2 unknown phosphorylated peptides when using MSA on the nano-LC-LTQ ion Trap instrument. Both phosphorylated peptides were manually validated and correspond to: R.VLVDQTTGLSR.G and R.SLFSSIGEVESAK.L. Those two phosphopeptides belong to the HuR RNA binding protein gi/1022961 protein. The analysis of 5 μl (~3 μg) of the sample purified by TiO_2 _and desalted and cleaned by R3/C18 permitted us to obtain 4 unknown phosphorylated peptides when using MSA on the nano-LC-LTQ instrument.

The four phosphorylated peptides were manually validated and correspond to: R.VLVDQTTGLSR.G, R.SLFSSIGEVESAK.L, K.DVEDMFSR.F which belong to the HuR RNA binding protein gi/1022961 and; another phosphopeptide: K.DLSSIFR.G which belongs to p38 MAP Kinase gi/1469306 (Table 1).

**Table 1:** The 3 phosphorylated proteins (HuR, Chain B and p38p) and the 6 phosphopeptides identified and validated (amino acid sequences below the identified proteins) when using SIMAC coupled to MAS by the LTQ ion Trap mass spectrometer are shown in this table.

Proteins	Phosphopeptides
HuR RNA binding protein gi/1022961	DVEDMFSphR (*)
	VLVDQTTphGLSR
	DANLYSphGLPR (*)
	SLFSSIGEVESphAK (*)

Chain B, Structure Of Appbp-1-Uba3-nedd8-Mgatp-Ubc 12 (c111a), A Trapped Ubiquitin-Like Protein Activation Complex gi/126031226	TphAVINAASGR (*)

P38 MAP Kinase gi/1469306	DLSphSIFR (*)

Around ≥ 3 μg of complex peptides mixture were loaded into SIMAC micro-columns and analyzed by nano-LC-ESI-LTQ ion Trap (Thermo).

In addition when using SIMAC coupled to DDNLMS3 3 proteins were identified: (a) HuR RNA binding protein gi/1022961, (b) P38 MAP Kinase gi/1469306 and (c) Change B, A Trapped Ubiquitin like-protein activation gi/126031226.

(a) From the phosphorylated protein HuR RNA binding protein gi/1022961, 3 phosphopeptides were isolated and validated (K.DVEDMFSR.F, K.DANLYISGLPR.T, R.SLFSSIGEVESAK.L).

(b) From the phosphorylated protein p38 MAP Kinase gi/1469306, one phosphopeptide was isolated and validated (K.DLSSIFR.G).

(c) From the phosphorylated protein Change B, A Trapped Ubiquitin like-protein activation gi/126031226, one phosphopeptide was isolated and validated (R.TAVINAASGR.Q).

[Identified and validated phosphopeptides using SIMAC coupled to DDNLMS3 are distinguished by the symbol (*)]

The analysis of the 5 μl (~3 μg) of the sample purified by SIMAC and desalted and cleaned by R3/C18 allowed us to obtain 6 unknown phosphorylated peptides when using MSA on the nano-LC-LTQ ion Trap instrument. The six phosphorylated peptides were manually validated and correspond to: K.DVEDMFSR.F, R.VLVDQTTGLSR.G, K.DANLYISGLPR.T, R.SLFSSIGEVESAK.L which belong to HuR RNA binding protein gi/1022961 and; R.TAVINAASGR.Q which belongs to Chain B, Structure Of Appbp1-Uba3-nedd8-Mgatp-Ubc12 (c111a), A Trapped Ubiquitin-Like Protein Activation Complex gi/126031226, and K.DLSSIFR.G which belongs to p38 MAP Kinase gi/1469306 (Table 1).

Therefore, when using MSA by the LTQ Ion Trap instrument, SIMAC (6 phosphopeptides purified, identified and validated) efficiency is higher than TiO_2 _(4 phosphopeptides purified and identified) and IMAC (2 phosphopeptides purified, identified and validated) for these protein-kinases studied. It has been described that IMAC easily enriches multiple phosphorylated peptides while TiO_2 _mono-phosphorylated ones. In fact, SIMAC has been optimized to get the best efficiency from IMAC and TiO_2 _and complement both in just one method (see reference previously mentioned [34]). This supports our data.

In any case, we recommend that in order to study kinase phosphorylated protein kinases, combine the three resins (or ever more phosphoenrichments methods) in order to purify as many as possible phosphopeptides [46]. The reason for this is that each sample needs to be optimized and tested with different complementary strategies. The analysis of the 5 μl (~3 μg) of the sample purified by SIMAC and desalted and cleaned by R3/C18 allowed us to get 5 unknown phosphorylated peptides when using Data Dependent Neutral Loss MS3 (DDNLMS3) on the nano-LC-LTQ ion Trap instrument. The five phosphorylated peptides were manually validated and correspond to: K.DVEDMFSR.F, K.DANLYISGLPR.T, R.SLFSSIGEVESAK.L which belong to HuR RNA binding protein gi/1022961; K.DLSSIFR.G which belongs to p38 MAP Kinase gi/1469306 and R.TAVINAASGR.Q which belongs to Chain B, Structure Of Appbp1-Uba3-nedd8-Mgatp-Ubc12 (c111a), A Trapped Ubiquitin-Like Protein Activation Complex gi/126031226 (Table 1).

**Figure 2:**
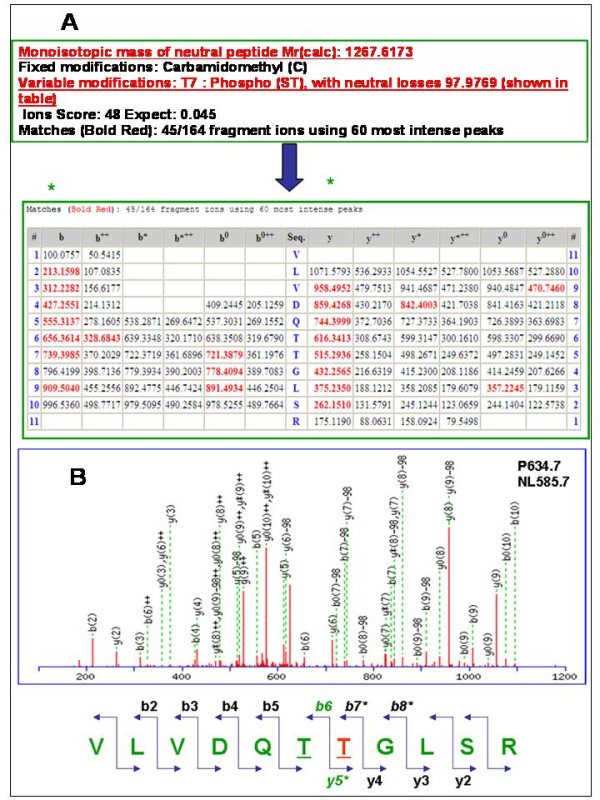
**Phospho-site assignment & manual validation of the phosphorylated peptide VLVDQTTphGLSR obtained by Mascot analysis**. The monoisotopic mass of neutral peptide Mr (calc) resulted was 1267.6173. Fixed modifications chosen were: Carbamidomethyl (C), while for variable modifications T7: Phospho (ST), with neutral losses 97.9769 (shown in table) was selected. The y5 ion and b7 are those which allowed identification of the treonine (5) as phosphorylated (ph) amino acid (T in red colour). In addition the phosphate fingerprint of the neutral loss (NL) from the parent ion is also a positive signal of phosphorylation. Six b ions and 8 y ions were continuously matched respectively.

(c) SIMAC coupled to MAS and MS3-NL mass spectrometry analysis. The preferred approach for analyzing samples using mass spectrometry is to produce structurally significant product ions using the process of ion dissociation. A method commonly known as Data Dependent Neutral Loss MS3 (DDNLMS3) (developed by Coon and co-workers [47]) analysis enables selective fragmentation by isolating a neutral loss ion fragment from an MS/MS experiment and then subjecting it to further dissociation [48]. Despite of this, DDNLMS3 did not allow us to get as efficient results as when using MSA for our protein-kinases analyses. It is well known that the production of neutral loss ions in MS/MS, is almost always accompanied by partial fragmentation of the precursor ion and these diagnostic fragment ions are subsequently lost when the neutral loss ions are isolated for MS3. Multistage activation (or pseudo MS3) allowed us to get spectra that were the combination of MS/MS and MS3 fragmentation and thus retaining the informative fragments from the precursor ion more efficiently. This is due to the fact that MSA produced more structurally informative ions by eliminating the ion isolation step between MS/MS and MS3 for the study of phosphorylated protein kinases p38 and HuR *in vitro*. We observed that - in this research study related to the previously phosphorylated proteins after *in vitro *kinase reaction- multistage activation was a faster route to a more information- rich spectra since the ion-trap does not require refilling for the MS3 scan, as with the traditional neutral loss experiment (DDNLMS3). We concluded during the first tests-analyses of the protein kinases *in vitro*, that when compared to DDNLMS3, multistage activation generated spectra with increased signal intensity and a greater number of structurally diagnostic ions for phosphorylated peptides. Thus we chose MSA as a routine path for this kind of analysis (p38 and HuR phosphorylated kinases *in vitro*). Further benefits of using multistage activation are demonstrated in other studies of phosphopeptides, including large scale analysis [49]. The information-rich spectra generated using multistage activation were particularly important for these compounds because there is often a significant loss of sequence informative fragment ions generated in MS/MS. For this study, more ions were identified with multistage activation than with MS/MS or MS3 in the DDNLMS3 method. In addition, the signal intensities were generally higher with multistage activation compared to MS/MS or MS3 of DDNLMS3 method. In fact, multistage activation resulted in more information for the suite of phosphopeptides studied (Table 1) (see an example of the spectrum of an identified phosphorylated peptide when using SIMAC coupled to MSA in the LTQ ion Trap mass spectrometer and Mascot, Figure 2).

**Figure 3:**
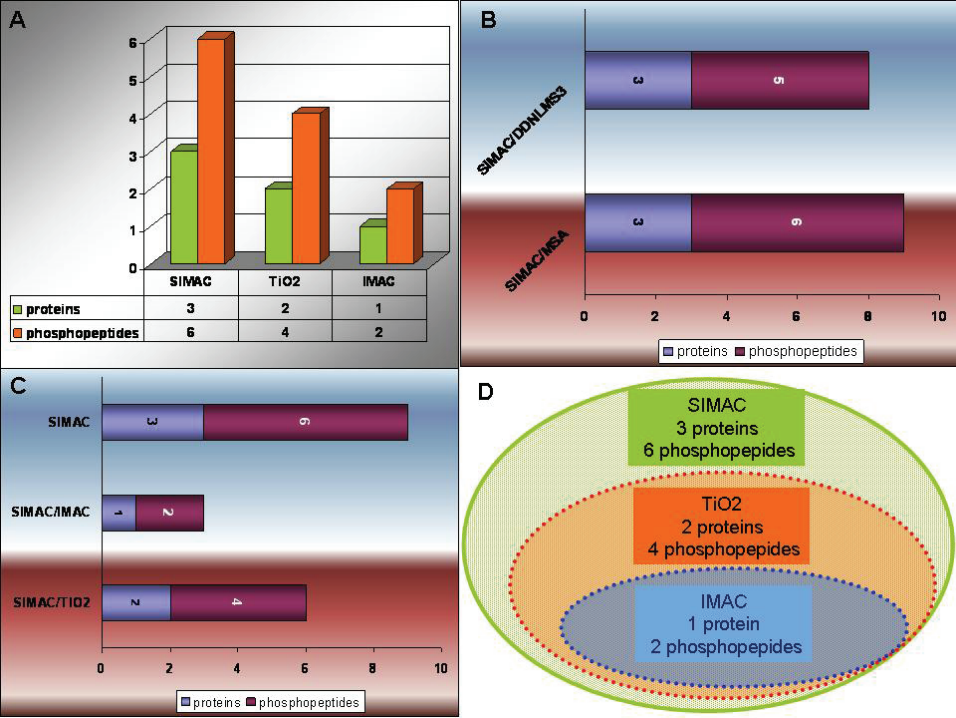
**The efficiency and reproducibility of the phosphopeptide purification and identification when using ~3 μg of protein kinases per each resin and/or phosphoenrichment method (SIMAC, TiO**_**2 **_**and IMAC) coupled to R3/C18 and MSA-LTQ ion Trap mass spectrometer is illustrated**. **[A] **Four triplicate experiments were carried out in order to identify the phosphopeptides. The phospho-site identifications were carried out from pooled and non-pooled assays (inter- and intra-assays) confirming a high reproducibility. The 6 phosphorylated peptides identified were isolated and validated in the four triplicate analyses, not only by Mascot (at least 4 continuously -y and -b ions matched)but also by manual inspection of all the spectra. SIMAC allowed the purification of 3 phosphorylated proteins: HuR RNA binding, p38 MAP Kinase and Trapped Ubiquitin-Like Protein Activation Complex, and 6 phosphorylated peptides related to those previously mentioned proteins. TiO_2 _and IMAC allowed the isolation of 2 phoshorylated proteins: HuR RNA binding and p38 MAP Kinase, and 1 phosphopeptide related to the protein kinase HuR RNA binding. **[B] **SIMAC coupled to MSA allowed the identification of one more phosphopeptide compared to SIMAC coupled to DDNLMS3. Nevertheless, both strategies (SIMAC coupled to MSA and SIMAC coupled to DDNLMS3) allowed the identification of the same number of phosphorylated proteins (3). **[C] **and **[D] **Three phosphorylated proteins and six phosphopeptides were identified when using SIMAC coupled to MSA. From those three phosphoproteins identified, six phosphopeptides were identified: (a) TiO_2 _coupled to MSA allowed the identification of two equal/same phosphorylated proteins and four equal/same phosphopeptides as SIMAC and (b) IMAC allowed the identification of one equal/same protein and two equal/same phosphopeptides. Thus, SIMAC is more efficient than the other tested resins for this study, while TiO_2 _and IMAC corroborate the reproducibility of the phosphorylated proteins and phosphopeptides identified.

Nevertheless, it must be pointed out that Jiang and co-workers developed a specific classification filtering strategy for their studies (using different samples) which significantly improved the coverage of the phosphoproteome analysis when using NLMS3 (see reference previously mentioned [48]). In fact, Jiang and co-workers obtained a higher coverage of the phosphopeptide identifications when processing and filtering specific methods which they developed for the spectra from NLMS3, compared with MS2 and MSA strategies. In relation to this, we should say that just one more phosphopeptide was identified and validated when we used SIMAC coupled to MSA (new 6 identified phoshopeptides) compared to when we coupled SIMAC to DDNLMS3 (5 new identified phosphopeptides). In addition, those 5 new phosphorylated peptides identified and their phospho-site assignments in each specific amino acid are the same ones following both strategies (see Figure 3 and Table 1). Moreover, the 6 new phosphopeptides and phospho-site assignments showed high reproducibility in all cases during the four triplicate experiments we carried out.

All our MS analyses were carried out by CID. We hypothesize that combining CID with ETD or ECD fragmentation, it is probable that more and/or complementary data would be obtained according to the methodological study of Navajas and co-workers [50]. ECD occurs only on the peptide backbone - which is an advantage -, and labile phosphate groups are left intact on the resulting c- and z- fragment ions, thus, complementary identification of other specific phosphorylation sites would be enabled [51,52]. As a result, we recommend using CID to start with, and would recommend switching to ETD, in the event you were not able to determine the phosphorylation site, if you have the possibility of the required instrument [53-57]. The phosphopeptides purified, identified and validated, including also the site-assignments of the phosphate group are illustrated in Table 1.

The efficiency and reproducibility of the phosphopeptide purification and identification when using ~3 μg of protein kinases per each resin and or phosphoenrichment method (SIMAC, TiO_2 _and IMAC) coupled to R3/C18 and MSA-LTQ ion Trap mass spectrometer is illustrated in Figure 3.

An example of a phospho-site assignment and manual validation of the phosphorylated peptide (VLVDQTTphGLSR) obtained by Mascot analysis is illustrated in Figure 2.

### Bioinformatic modelling and molecular dynamics simulations

**Figure 4:**
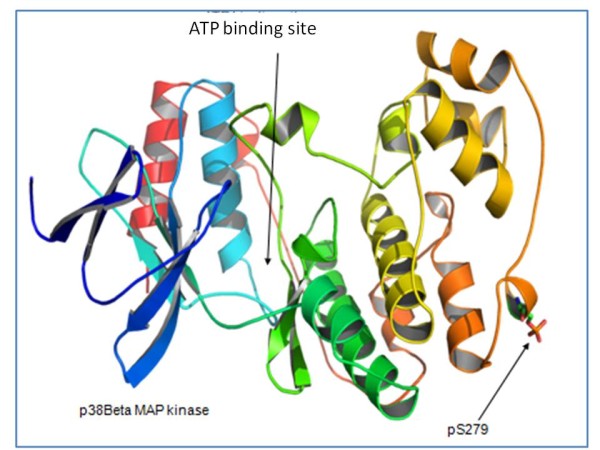
**Location of phosphorylated Ser-279 in the protein structure of human MAP kinase p38beta (p38B)**. A model for phosphorylated serine was located in the structural position of residue Ser-279 in the 3D crystallographic coordinates of p38B (Protein Data Bank code: 3GC8). Position of the ATP binding site is indicated. Plot was generated using PyMOL (DeLano Scientific, San Carlos, CA).

To study the potential functional effect of serine phosphorylation in the above indicated sequence locations, 3D structural models for the phosphorylated state of both MAP kinase p38beta (p38B) and HuR were generated using bioinformatics procedures. As shown in figure 4, phosphorylated Ser-279 of p38B is located in a loop placed on the external surface of the protein structure, far away from the active site of the kinase. It is conceivable that the phosphorylation of this residue does not affect p38B structure stability or folding, but external contacts to accompanying proteins, modulate the nature of the putative interaction (see reference previously mentioned [39]).

In the case of HuR, only one of the four phosphorylated residues found (Ser-48) fall into a structure domain of the protein previously crystallized: the dimmerized first RNA recognition motif (see reference previously mentioned [40]). Thus, only the putative structural effect of the phosphorylated and non-phosphorylated state of Ser-48 could be analyzed through structural bioinformatic tools including Molecular Dynamics (MD) simulation.

**Figure 5:**
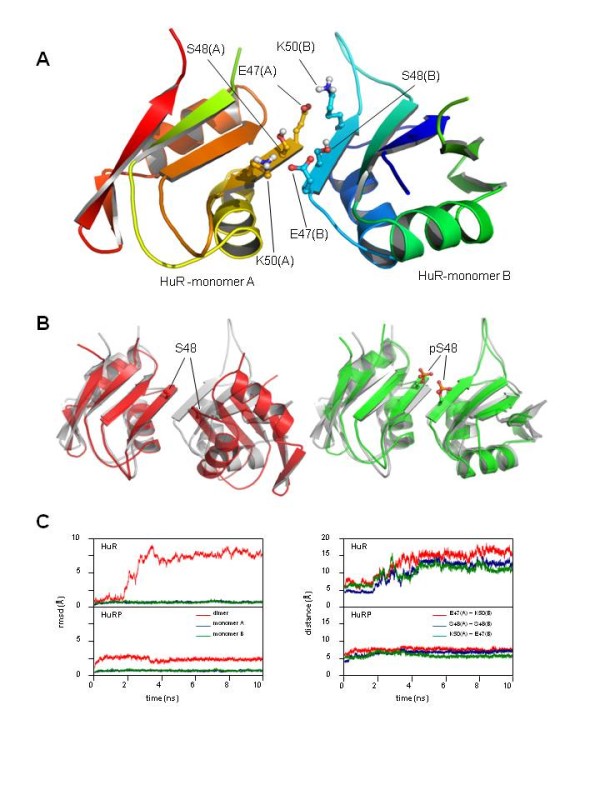
**Effect of the phosphorylation of Ser-48 in the stability of the dimmer structure of the first RNA recognition motif of HuR**. **[A] **Crystal structure of HuR dimmer (Protein Data Bank code: 3HI9, chains B and D) indicating the position of Ser-48, Glu-47 and Lys-50 in the dimmerization surface. **[B] **Relative spatial position of the two HuR monomers after 10ns of unrestricted Molecular Dynamics (MD) simulation of both non-phosphorylated (red structure, left) and phosphorylated (green structure, right) states of Ser-48. Position of initial dimmer structure, prior to the MD simulation, is included for comparison (in gray). Note the large displacement of the non-phosphorylated state in contrast to the stability exhibited by the dimmer in presence of phosphoSer48. Respective positions of Ser-48 and phosphoSer-48 are indicated. **[C] **Left: RMSD values measured for HuR dimmer (red) monomer A (blue) and monomer B (green) during the 10 ns trajectory of the unrestricted MD simulation of the dimmer in presence of non-phosphorylated Ser-48 (HUR plot, top panel) or phosphorylated pSer-48 (HURP plot, lower panel). Right: continuous measurement of Cα Cα distances between residues E47(A)-K50(B) (red), S48(A)-S48(B) (blue) and E47(A)-K50(B) (green), during the MD trajectory. Distortion of the HuR dimmer in the presence of Ser-48 (HUR plot, top panel) when compared to the phosphorylated state of the protein (HURP plot, lower panel) is patent in both RMSD and Cα-Cα measurements. Structure plots were generated as in Figure 4.

As shown in figure 5A, Ser-48 is located in the dimmerization surface, surrounded by residues Glu-47 and Lys-50 that form a pair of saline bonds potentially implicated in the stabilization of the dimmer. To test the effect of the presence of a phosphorylated Ser in the maintenance of the quaternary structure, two unrestricted MD computer simulations were performed in presence or absence of a phosphorylated Ser in position 48, as indicated under "Materials and Methods". Results obtained after 10ns of MD showed that, in the case of the unphosphorylated dimmer structure (non-phosphorylated Ser-48), both monomers experimented a significant displacement from their initial relative positions, resulting in a complete disorganization of the quaternary structure (Figure 5B -left-). Measurement of root mean square deviation (RMSD) values of the monomers and the dimmer, as well as the distances between the C atoms of the contact residues (Glu47A-Lys50B, Ser48A-Ser48B and Lys50A-Glu48B) indicated a clear and irreversible displacement from the initial values during the first steps of the MD simulation (Figure 5C, upper plots). In contrast, when the same MD simulation was performed in presence of a phosphorylated Ser in position 48 of both monomers, a clear stabilization of the dimmerized structure was obtained, showing no displacement from their initial position (Figure 5B -right-) and exhibiting constant values of RMSD values and invariable RMSD values and distances between contact residues (Figure 5C, lower plots).

These results suggest that the phosphorylation of Ser48 in the first protein RNA recognition motif of HuR has potentially a stabilizing effect, exerting a regulatory role on the biological function of protein by regulating the maintenance of the dimmerized quaternary state, a requirement of the complex prior to RNA binding activity [58].

## Discussion

Reversible protein phosphorylation plays an important role in the regulation of many different processes, such as cell growth, differentiation, migration, metabolism, and apoptosis. Identification of differentially phosphorylated proteins by means of phospho-proteomic analysis coupled to bioinformatic tools provides insight into signal transduction pathways that are activated in response to, for example, growth factor stimulation or toxicant-induced apoptosis [59]. Four triplicate experiments were carried out in order to identify the phosphopeptides of the protein kinases studied in this article (p38 and HuR). The phospho-site identifications were carried out from pooled and non-pooled assays (inter- and intra-assays) confirming a high reproducibility: the 6 isolated and identified phosphorylated peptides were validated in the four triplicate analyses by Mascot (score >20, and at least 4-y and -b ions continuously matched) also including manually inspection via phosphate-fingerprints of all the spectra. It should be pointed that some spectra showed a better resolution than others. Three different resins (IMAC, TiO_2 _and SIMAC) were used despite the fact that it is well known that SIMAC has been developed and described as a method to purify more phosphopeptides for high-throughput analysis giving great efficiency (see references previously mentioned [34-36]). But it has also been demonstrated that the analyses of different phosphorylated proteins when using different resins (*e.g*. TiO_2_, IMAC or ZrO_2_) -including SIMAC- are more efficient in a complementary way for specific samples. This is due to the fact that the intrinsic characteristic of each protein (*e.g*. amino acid composition). In fact, in order to get as many phosphopeptides as possible from the same sample, different tests must be carried out [*e.g *of other possibilities of phosphoenrichments: Strong cation and anion exchange (SCX and SAX), Calcium phosphate precipitation, Hydrophilic interaction chromatography (HILIC)]. Moreover, phosphoproteomic analysis of kinases implies more difficulties as they usually are low expressed proteins with the disadvantage that the un-phosphorylated form is more abundant than the phosphorylated within the same sample to be analyzed. In addition, IMAC elutes easily multi-phopshorylated peptides than TiO_2 _and ZrO_2_. TiO_2 _and ZrO_2 _have the capacity of binding strongly the multiple phosphorylated peptides, thus both last mentioned resins retain multi-phosphorylated peptides and do not give -usually- the chance to elute them. We chose TiO_2 _for our research study instead of ZrO_2 _although they are of similar characteristics, but further experiments using ZrO_2 _or other resins or different possibilities of phosphoenrichments (SCX and/orHILIC for *e.g*) could surely give complementary and interesting data for signalling network research advances. The phosphopeptides found in the analyzed sample corresponding to p38 MAP kinase protein do not include the described phosphorylation sites in the T-loop (pThr-180 and pTyr-182). We hypothesize that the protein was not phosphorylated in those precise sites in the original sample, indicating a particular inactive state of the kinase activity [60,61]. In addition, we detected unphosphorylated peptides after protein digestions by MS and Mascot analysis. These unphosphorylated peptides were very useful for the assured identification of the studied protein kinases. During the evaluation of the data, we found that the peptide scores of phosphopeptides assigned by Mascot did not always correlate with the quality of the spectra, and for this reason, all fragmentation spectra were manually verified, in order to avoid false positives. A peptide was accepted when at least four consecutive y- and b- ions were assigned to abundant signals in the fragmentation spectra. If this was not the case, then the amino acid sequence was inspected to look for indications of why these y/b-ions were missing. Peptides lacking a C-terminal arginine or lysine residue, due to a C-terminal position in the particular protein sequence, were accepted, when at least four consecutive b-ions had been assigned in the fragmentation spectra. Peptides with a C-terminal lysine residue and an N-terminal arginine residue due to missed cleavage were also accepted, when at least four consecutive b-ions were assigned in the fragmentation spectra. In addition, the spectra were inspected for the presence of proline residues, which usually give rise to intense signals, suppressing other signals in the spectra. A neutral loss of 98 Th due to the loss of phosphoric acid resulting from gas-phase Β-elimination of phosphoserine or phosphothreonine residues together with the presence of dehydroalanine or dehydro-2-amino butyric acid or the presence of intact phosphoserine or phosphothreonine residues in the sequence were used as indicators of a serine or threonine phosphorylated peptide, respectively. The characteristic phosphotyrosine immonium ion at 216.05 Th or the presence of an intact phosphotyrosine residue in the sequence was used for confirmation of tyrosine phosphorylation if it was the case. The analysis by the LC-nano-ESI-LTQ ion Trap and the manual validation of the MS/MS and MSA spectra was performed by EL following the rules of Mann and Jensen (see references [36,57]). (Table 1, Figure 2, Figure 3).

One of the disadvantages of Electron Capture Dissociation (ECD) is that it has selectivity for disulfide bonds, due to the high radical affinity of the bond. Further analysis related to these protein kinases will be carried out as it preserves the intact information about labile modifications, which are not observed directly when using CID at the same time with the knowledge that the drawback of ETD is less sensitive compared to CID, because of lower ionization efficiency. Thus more complementary data would be available soon. A third phosphorylated protein (Trapped ubiquitin-like protein complex gi/126031226) was isolated and identified during the MS analysis of the p38 and HuR protein kinases. Apparently, this third phosphorylated protein seems to be linked to p38 and HuR kinases. The reason for that could be the kinase assay. The origin of the Ubiquitin-like protein activation complex protein needs to be studied in a deep way, as although p38/MAPK is a fundamental actor for the network connectivity of signalling partners, no data yet clearly implicate gi/126031226 in these processes or interactions (see references [53-59])

MD bioinformatics simulations are often good indicators of potential behaviour of protein-protein complexes; but they are only computational results, but coupled to experimental proteomics data, they give relevant clues. In fact, this study suggests that the phosphorylation of Ser48 in the first protein RNA recognition motif of HuR has potentially a stabilizing effect (Figure 5), exerting a regulatory role on the biological function of protein by regulating the maintenance of the dimmerized quaternary state, a requirement of the complex prior to RNA binding activity (see reference [58]). In addition, a figure illustrating the 3D position of phosphorylated Ser-279 in the structure of human MAP kinase p38beta (Figure 4) (p38B) indicates clearly that this precise phosphorylation site is located far from the ATP binding site and also far from the T-loop, indicating a different role for this residue, which opens a new research door for this relevant protein kinase [59-61].

## Conclusions

Our proteomics studies have demonstrated that there are 6 new phosphopeptides of the protein kinases p38 and HuR during *in vitro *assays. This was possible by testing different resins coupled to different MS strategies to isolate and identify phosphopeptides. The identified phosphopeptides were manually validated and the phospho-site assignments were also carried out in order to avoid false positives. Recently, several signalling studies for clinical research are ongoing and each sample needs to be tested by different strategies in order to check which one adapts better to your goals, the characteristics of the proteins to be studied, and the possibility of getting complementary data. The experiments carried out showed excellent reproducibility in the four triplicate experiments for the identification and validation of the identified phosphopeptides, also for the protein identifications of p38 and HuR kinases. A Trapped ubiquitin-like protein complex gi/126031226 was also isolated and identified containing one phosphopeptide during this study. The origin of the Ubiquitin-like activation complex protein needs to be studied in a depth as although p38/MAPK is a fundamental actor for the network connectivity of signalling partners, no data yet clearly implicate gi/126031226 in these processes or interactions. Overall, the combination of SIMAC and MSA resulted more efficient to isolate and identify phosphopeptides from p38 and HuR protein kinases. It should be pointed out that when using other phosphoenrichment alternatives (SCX and HILIC) coupled to other MS strategies for this study, we will be able to corroborate even more complementary data related to the results presented.

Bioinformatic studies, using structural tools, of reversible phosphorylation in proteins will allow the generation of useful models for protein-protein contacts at the atomic level. These models can be used as input for sophisticated techniques, as molecular dynamics, that offer the possibility of analyzing the potential effect of the phosphorylation and de-phosphorylation process of each residue in the global structure and behaviour of the protein. In the present work, the molecular dynamics analysis of the phosphorylation of Ser-48 in HuR homodimmer offered a suitable hypothesis of how the phosphorylation state of Ser-48 can regulate the protein mechanism through controlling the homodimmerization arrangement of the corresponding structural domain. Regarding the 3D position of phosphorylated Ser-279 in the structure of human MAP kinase p38, molecular simulation clearly indicates that this precise phosphorylation locus is located far from the ATP binding site and also far from the T-loop, indicating a different role for this residue: this could open a new and relevant research door for this important protein kinase, which has been related as a connectivity-link for different signalling pathways.

## List of Abbreviations

**AQUA**: Absolute Quantification; **CID**: Collision-Induced Dissociation; **Da**: Dalton (molecular mass); **DIGE 2-D**: Fluorescence Difference Gel Electrophoresis; **ECD**: Electron Capture Dissociation; **ESI**: Electron Spray Ionization; **ETD**: Electron Transfer Dissociation; **FT-ICR**: Fourier transform-Ion Cyclotron Resonance; **HILIC**: Hydrophilic interaction chromatography; **HPLC**: High-performance liquid chromatography or high-pressure liquid chromatography; **H**_**3**_**PO**_**4**_: Phosphoric acid; **ICR**: Ion Cyclotron Resonance; **IMAC**: Immobilized Metal Affinity Capture; **IT**: Ion Trap; **iTRAQ**: Isobaric Tag for Relative and Absolute Quantification; **kDa**: kilodalton (molecular mass); **LC**: Liquid Chromatography; **MALDI**: Matrix-Assisted Laser Desorption/Ionization; **MD**: Molecular Dynamics; **MOAC**: Metal Oxide Affinity Chromatography; **Mr**: Relative molecular mass (dimensionless); **MRM**: Multiple reaction monitoring; **MS**: Mass Spectrometry; **MSA**: MultiStage Activation; **MS/MS**: tandem mass spectrometry; **m/z**: Mass to charge ratio; **PID**: Primary Immunodeficiencies; **PTM**: Post-Translational Modification; **SILAC**: Stable Isotope Labelling with Amino acid in cell Culture; **SIMAC**: Sequential Elution from IMAC; **TiO**_**2**_: Titanium dioxide; **TOF**: Time Of Flight; **ZrO**_**2**_: Zirconium dioxide.

## Competing interests

The authors declare that they have no competing interests.

## Authors' contributions

Author 1 (EL) carried out the proteomics, phosphoproteomics and mass spectrometry studies for this article. Authors 2, 3, 4 (IL, JS, AF) carried out the clinical studies in order to support this article. (Contributions of PGP and RM) support the bioinformatic section coupled to proteomic, special thanks for PGP, RM and ARN for making possible the publication of this article. All authors read and approved the final manuscript.
